# Age-Related Diseases and Foods Generating Chlorinative Stress

**DOI:** 10.3390/antiox12020249

**Published:** 2023-01-22

**Authors:** Eleonora Di Salvo, Marco Casciaro, Concetto Mario Giorgianni, Nicola Cicero, Sebastiano Gangemi

**Affiliations:** 1 Department of Veterinary Sciences, University of Messina, 98168 Messina, Italy; 2 School and Unit of Allergy and Clinical Immunology, Department of Clinical and Experimental Medicine, University of Messina, 98125 Messina, Italy; 3 Department of Biomedical, Dental and Morphological and Functional Imaging, University of Messina, 98125 Messina, Italy; 4 Science4life srl, Spin off Company, University of Messina, 98100 Messina, Italy

**Keywords:** oxidative stress, chlorinative stress, hypochlorus acid, aging, old, elderly, food, pesticides, diet

## Abstract

Background: Aging is a slow and inexorable process affecting all life beings and is characterised by age-related worsening in adaptation to external changes. Several factors contribute to such a process, and oxidative stress due to external damages is one key player. Of particular interest is the oxidative stress generated from halogen compounds such as chloride. Hypochlorus acid is produced starting from MPO’s interaction with hydrogen peroxide. We focused on the oxidation of tyrosine residues by HOCl, which leads as a result to the formation of 3-chlorotyrosine (3-ClTyr). This molecule, due to its stability, is considered a marker for MPO activity. Results: We collected data from literature research articles evaluating chlorinative stress and the effects of 3-ClTyr on chronic diseases linked to aging. As diseases are not the only source of 3-ClTyr in people, we also focused on other origins of chlorinative stress, such as food intake. Discussion: Oxidation and halogenation are caused by infectious diseases and by pathologies characterised by inflammation. Moreover, diet could negatively or positively influence chlorinative stress. Comparing 3-ClTyr levels in the oldest and youngest old with age-related diseases and comparing data between different geographic areas with different pesticide rules could be the next challenge.

## 1. Introduction

Aging is a slow and inexorable process affecting all life, being characterised by an increasing worsening in adaptation to external changes and in a growing difficulty to maintain body homeostasis due to a diminished renewal capacity by cells and tissues. Several factors contribute to such a process, and oxidative stress due to external damages is one key player. Oxidative stress can originate from an exaggerated response to biological agents, such as in infectious diseases. In fact, in the attempt to counteract external pathogens, the oxidative burst of immune system cells can become harmful. Reactive oxygen species (ROS), highly unstable oxidating molecules, can also be generated by a lack of antioxidant systems in chronic diseases such as diabetes, COPD, and autoimmune disease [1,2,3]. Of particular interest is the oxidative stress generated from halogen compounds such as chloride (Cl^−^).

**Figure 1:**
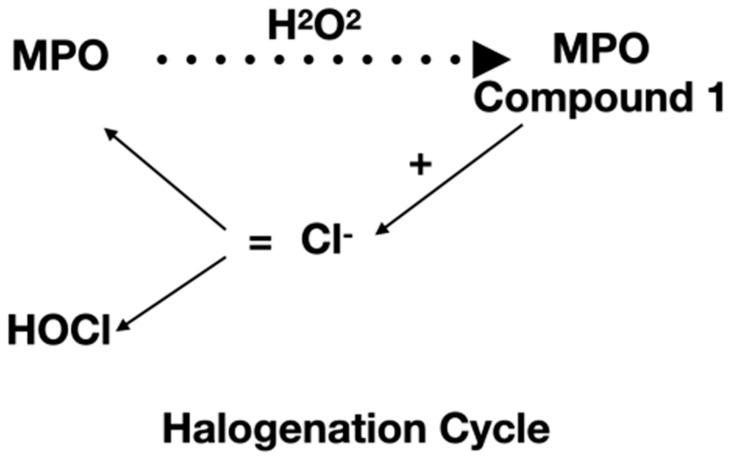
Hypochlorus acid production starts from MPO interactions with hydrogen peroxide.

During the life span, the human being undergoes inflammatory processes that involve myeloperoxidase (MPO) as a defense system. MPO has a weak antimicrobial activity, which is potentiated by the interaction with hydrogen peroxide. Native MPO is converted to MPO compound I, which can in turn start the “halogenation cycle” or the “peroxidation” one. Once the halogenation cycle is started, MPO compound I undergoes a two-electron reduction with halides/pseudohalides such as chloride (Cl^−^) to produce hypochlorous acid [4]. Instead, with the peroxidation cycle, MPO compound I, forming a MPO compound II complex, encounters two consecutive one-electron reductions, resulting in several radicals [5] (Figure 1).

MPO can work as a tool for many chemical reactions. Although it has such interchangeability, its main role is to produce hypochlorous acid (HOCl). HOCl is a powerful oxidating agent for biological agents. It can modify many biomolecules, such as DNA, lipids, and lipoproteins [6]. Of course, as an ROS, HOCl cannot stably maintain its form, so it interacts quickly with atoms of sulphur and nitrogen (nucleophile groups), typically present in thiols and thioethers (in cysteine and methionine residues), amines, and amides [7]. These interactions with thiol groups can deeply modify the biomolecules. The inactivation or activation of cellular enzymes such as metalloproteinases (MMPs) or proteinases can alter signalling cascades, compromising cell and tissue homeostasis [8]. Equally, the modification of amines and amides by HOCl generates chloramines and chloramides with detrimental cellular consequences.

**Figure 2:**
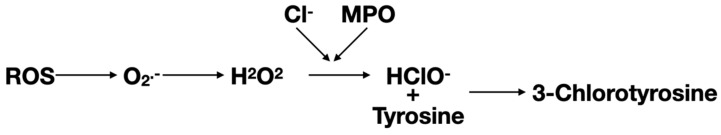
Hypochlorous acid is formed from hydrogen peroxide and chloride anion by MPO as a catalyst. MPO catalyses the transformation of hydrogen peroxide (H^2^O^2^) and chloride anion (Cl^−^) into highly reactive hypochlorous acid. 3-ClTyr is a product resulting from the combination of HOCl and *p*-tyrosine.

Here, we focus on the oxidation of tyrosine residues by HOCl, which leads as a result to the formation of 3-chlorotyrosine (3-ClTyr). This molecule, due to its stability, is considered a marker for MPO activity (Figure 2) [9,10,11,12].

As mentioned above, 3-ClTyr is easy to detect in serum due to its stability, and reflects the oxidative stress status linked to MPO activity and HOCl presence. However, it is also responsible for a direct influence on human homeostasis, as reported by some authors on the cardiovascular system. MPO-mediated chlorination of HDL impairs the direct binding of HDL to the ATP-binding cassette transporter (ABCA1) responsible for removing the exceeding intracellular cholesterol from macrophages. As a direct consequence, chlorination contributes to atherogenesis. Moreover, free 3-ClTyr can lead to direct vascular lesion formation by promoting the migration of human aortic smooth muscle cells [13].

The action of 3-ClTyr on the cardiovascular system is only one of the potential detrimental effects of the molecule on human pathophysiology. For this reason, we decided to collect data from literature research articles evaluating chlorinative stress on and the effects of 3-ClTyr on chronic disease linked to aging. As diseases are not the only source of 3-ClTyr in people, we focused also on other origins of chlorinative stress, such as food intake.

## 2. Materials and Methods

We spanned literature from inception until 31 October 2022. We entered the terms “aging”, “elderly”, “oldest old”, “centenarians”, “nonagenarians”, and “octagenarians” together with “chlorinative stress”, “3-chlorotyrosine”, “HOCl^”^, “chlorine”, and “hypochlorous acid”. We selected only articles that were in English. First, we excluded articles whose title did not match with our topic. Then, we read the abstract and then the entire paper of the research articles, focusing on the role of chlorinative stress in patients aged 65 or above.

## 3. Results

A total of 13 research articles evaluating the correlation between chlorinative stress and age-related disease were collected from the literature (Table 1).

### 3.1. Neurodegenerative Diseases

Pena-Bautista et al. studied 53 patients with a mean age of 70 affected by Alzheimer’s. Patients’ urine levels did not show any significant differences regarding tyrosine chlorination (3-Cl-Tyr/p-Tyr) vs. age-matched healthy controls. The authors speculated that the chlorination product levels were below the limit of detection since the experiment was conducted on urine samples [14].

Garcia-Moreno et al. conducted their analysis on 60 patients affected by Parkinson’s disease with a mean age of 63. In these patients, 3-chlorotyrosine was not detected nor in serum or in cerebrospinal fluid [15].

### 3.2. Chronic Obstructive Pulmonary Disease (COPD)

O’Donnel et al. reported 3Cl-Tyr levels in 14 patients with a mean age of 65 affected by COPD. Their study presented some criticism, because in healthy patients it was particularly difficult to collect spontaneous sputum, with them being “healthy”. However, they demonstrated an intimate link between 3Cl-Tyr, neutrophil number, and MPO activity. Their data suggested a positive correlation between 3Cl-Tyr and lung inflammation [16].

### 3.3. Kidney Failure (KD)

Afshinnia et al. noticed a positive correlation between 3Cl-Tyr levels and a worse chronic KD stage in 23 patients with a mean age of 67. They also reported an independent link with coronary artery disease [17].

The association with kidney failure was further confirmed by Delporte et al., who reported 3Cl-Tyr augmented levels in 15 haemodialysis patients aged 77 [18].

### 3.4. Coronary Disease (CD)

Cheng et al. evaluated 77 patients with acute CD with a mean age of 65. 3-Cl-Tyr levels were importantly increased in patients with an acute myocardial infarction (MI) compared to normal controls [19].

Wang et al. did not find differences in HDL-3-ClTyr levels between a group of 20 patients aged 63 and healthy controls [20].

A similar trend was confirmed by research on serum on a group of 512 patients affected by MI with a mean age of 62 [21].

Shao et al. reported elevated serum 3-ClTyr in 20 patients with stable coronary disease aged 64 and in 20 patients with acute coronary syndrome aged 63 [22].

### 3.5. Colorectal Cancer (CRC)

A positive correlation of 3-ClTyr with CRC and with cancer stage was found in 75 patients with a mean age of 66 [23].

### 3.6. Rheumatoid Arthritis (RA) and Vasculitis

Stamp et al. reported that 3-ClTyr values in a group of 77 patients aged up to 82 years old were significantly augmented [24].

Another study by Vivekanandan-Giri et al. confirmed the trend, although the mean age in the 38 patients analysed was 63 [25].

Higashi et al. demonstrated a significant increase in 3-ClTyr in urine during the acute phase of the disease in eight patients affected by vasculitis with a mean age of 66 [26].

### 3.7. Brain Levels

Kato et al. analysed chlorinative stress in the aged brain in three patients with a mean age of 69 affected by aneurism, myocardial infarction, and lung cancer, respectively. In every case, 3-ClTyr was detectable [27].

## 4. Discussion

As discussed above, MPO activity and HOCl formation is a physiological event that affects all human beings. The main purpose of HOCl’s presence is its antimicrobial function. As happens for other ROSs, their presence is counterbalanced by a scavenging system and by the intervention of antioxidant body defences, which are mainly enzymatic. Chlorinative stress is a result of MPO activation. In every age-related disease mentioned above there is the activation of such an enzyme. Neutrophils, together with specific monocyte–macrophage line cells activate MPO and produce HOCl. MPO is a glycosylated haem enzyme deposited in neutrophils and macrophagous azurophilic granules; these granules have a powerful bactericidal action, which is due to the production of hypochlorous acid from hydrogen peroxide and chloride ions. MPO could be released extracellularly; augmented serum levels of MPO have been described in chronic inflammatory conditions typical of age-related diseases such as atherosclerosis, neurodegenerative conditions, and some cancers [28,29,30,31]. Oxidative and chlorinative stress are key features of aging. Age-related diseases are characterised by high ROS levels [32,33]. What distinguishes successful aging is the ability of the person not to undergo the oxidative stress and inflammation without counterbalancing it. It can happen that the oldest elderly have a coexisting condition of high inflammatory parameters but also an optimal anti-inflammatory counterbalance, guaranteeing long-lasting healthy status [34].

As we reported by spanning the literature, oxidation and halogenation are caused by infectious disease, but also happen in pathologies characterised by intense inflammatory status. A young subject can more easily face elevated ROS levels in cardiovascular, metabolic, or autoimmune diseases. As we report, often, aged people with such diseases suffer more chlorinative stress, which is testified by elevated 3-ClTyr levels. This compound could modify the proteins’ structure, compromising their optimal function. This is a worsening factor in such already detrimental diseases. Whichever is the source of HOCl, the result is more oxidative and chlorinative stress, and in turn, more inflammation. For this reason, reducing the levels of HOCl is one important step in reducing inflammaging. This goal can be reached in diverse manners. The lifestyle is one of the best options in a long-term period and is a variable of ease intervention by the patient itself.

### Chlorinative Stress: Food, Help, or Threat?

In recent years, the close correlation between chlorination and diet has been demonstrated. Nevertheless, the use of chlorinated substances has been restricted in the instance of the Global Stockholm Convention (UNEP, 2004), both in Europe and the USA [35]. Until then, the most commonly used chlorinated substances were organic pesticides, used in the agrifood industry, such as dichlorodiphenyldichlorethylene (DDE), hexachlorbenzene (HCB) [36], and persistent organic pollutants (POPs). This set of toxic chemicals are persistent in the environment and able to last for several years because they accumulate in the fatty tissue of animals and humans, as well as in the ground [37]. Although the use of these substances has been forbidden, some studies have reported their presence in human tissues several times [38]. Children and foetuses are no exceptions [39]. However, epidemiologic [40] data demonstrated that the central nervous system is one of the targets of polychlorinated biphenyls (PCBs). The early or midlife brain intake of PCBs worsens nervous tissue aging, with an augmented risk of neurodegenerative disease [41]. Furthermore, exposure to these chemicals has been shown to increase risk based on age, causing adverse reproductive outcomes that may be related to exposure to POPs, and consequently altering oxidative stress markers [42,43]. Although regulated pesticide levels seem to be decreasing, there have been traces of DDT and other chlorinated compounds in recent studies conducted in Italy and Spain. Unfortunately, there is an obvious difficulty in comparing the variations in exposure in various countries because the studies refer to the total concentrations of the chlorinated substance in the food product rather than in the fat or liver of animals or fish.

On the other hand, vegetarian food could be a source of antioxidant molecules, serving as nutraceuticals. In fact, the abovementioned pesticides have been banned from most industrialised countries. Only food originating from remote areas could be a source of chlorination. In contrast, some substances present in food can help to counteract the damage caused by chlorinated chemicals, modulating the immune system and inhibiting the action of oxidative stress. Food and physical exercise demonstrated their potential in ameliorating oxidative stress status. Only few data were reported about gender differences in ROS production response. Mattioli et al. focused on these differences, noticing a diversity in food intake response between women and men [44]. One of the most promising nutrients in terms of its antioxidant capacity is resveratrol [41,45]. Moreover, substances extracted from plant material, such as fruit rich in anthocyanins, flavonoids and total polyphenols [46]; oil [47]; and chicken meat reformulated by the partial replacement of meat by Mediterranean plant ingredients [48], have been proven to have antioxidant qualities and health benefits. We have listed some of the foods acting as nutraceuticals with the molecules cited above (Table 2).

## 5. Conclusions

Most of the data obtained by several authors independently demonstrate that the trend in 3-ClTyr concentration in age-related disease and in inflammatory conditions is elevated. As mentioned above, ROSs are a paradigm of inflammation, which is the main characteristic of aging. For this reason, the term “inflammaging” was elaborated. Reaching old age is determined mainly by successful aging, which is typical of subjects with specific characteristics together with a favourable lifestyle. In a previous study [51] and in this paper, we reported that chlorinative stress is present in many diseases typical of the elderly. We also reported how diet could negatively or positively influence chlorinative stress, building the basis for a comparison of 3-ClTyr levels in the oldest old vs. youngest old, both with age-related disease and in healthy controls. Comparing the data between different geographic areas and countries could also give a scenario about the influence of pesticides in such results.

## Figures and Tables

**Table 1:** A list of the research articles included in the review evaluating chlorinative stress in elderly patients.

Reference	N° of Patients	Mean Age	Tissue	Disease	3-ClTyr
[14]	53	70	Urine	Alzheimer’s disease	Normal
[15]	60	63	Serum; Cerebrospinal fluid	Parkinson’s disease	n.d.
[16]	14	65	Sputum	COPD	+
[17]	23	67	Serum	Kidney failure	+
[18]	15	77	Serum	Kidney failure	+
[19]	77	65	Serum	Acute myocardial infarction	+
[20]	20	63	Serum	Coronary disease	Normal
[21]	512	62	Serum	Myocardial infarction	Normal
[22]	20	64	Serum	Stable coronary disease	+
[22]	20	63	Serum	Acute coronary syndrome	+
[23]	75	66	Serum	Colorectal cancer	+
[24]	77	82	Serum	Rheumatoid arthritis	+
[25]	38	63	Serum	Rheumatoid arthritis	+
[26]	8	66	Urine	Vasculitis	+
[27]	3	69	Brain	Aneurism; myocardial infarction; lung cancer	+

**Table 2:** Examples of foods rich in polyphenols helpful in reducing oxidative stress, typical of the Mediterranean diet.

Foods Rich in Polyphenols [49,50]
Strawberries
Raspberries
Cherries
Blueberries
Grapes
Mulberries
Olive oil

## Data Availability

Not applicable.
